# Evaluation of Angiogenesis Assays

**DOI:** 10.3390/biomedicines7020037

**Published:** 2019-05-16

**Authors:** Zachary I. Stryker, Mehdi Rajabi, Paul J. Davis, Shaker A. Mousa

**Affiliations:** 1 Department of Health, Division of Environmental Health Sciences, Albany, NY 12237, USA; zachary.stryker@health.ny.gov; 2 The Pharmaceutical Research Institute, Albany College of Pharmacy and Health Sciences, Rensselaer, NY 12144, USA; mehdi.rajabi@acphs.edu (M.R.); pdavis.ordwayst@gmail.com (P.J.D.); 3 Department of Medicine, Albany Medical College, Albany, NY 12208, USA

**Keywords:** angiogenesis, pro-angiogenesis, anti-angiogenesis, anticancer, growth factors, integrin αvβ3, vascular endothelial growth factor (VEGF), vascularization, endothelial cells

## Abstract

Angiogenesis assays allow for the evaluation of pro- or anti-angiogenic activity of endogenous or exogenous factors (stimulus or inhibitors) through investigation of their pro-or anti- proliferative, migratory, and tube formation effects on endothelial cells. To model the process of angiogenesis and the effects of biomolecules on that process, both in vitro and in vivo methods are currently used. In general, in vitro methods monitor specific stages in the angiogenesis process and are used for early evaluations, while in vivo methods more accurately simulate the living microenvironment to provide more pertinent information. We review here the current state of angiogenesis assays as well as their mechanisms, advantages, and limitations.

## 1. Introduction

Angiogenesis is the formation of new blood vessels from the endothelial cells (ECs) of pre-existing veins, arteries, and capillaries, and is essential for the progression of many cancers and other pathological states. The feasibility of clinically modulating angiogenesis was convincingly first shown by Judah Folkman [1,2]. Because angiogenesis plays a significant role in ischemic disease and in the metastases of many cancerous tumors, the pro- or anti-angiogenic effects of certain pharmaceuticals can be applied to effectively treat many of these pathologies. Therefore, angiogenesis assays have been devised, with the primary objective of determining which biomolecules operate most effectively and efficiently on altering angiogenesis processes in human subjects.

Angiogenesis plays a prominent role in cancer metastasis in particular, representing one of the major areas of cancer research in recent years [2,3,4,5]. Tumors can induce angiogenesis through the release of vascular endothelial growth factor (VEGF) and basic fibroblast growth factor (bFGF), which act as promoters of new blood vessel formation [6,7]. ECs respond to pro-angiogenic biomolecules and expand the existing vascular structure to reach the tumor so that the tumor cells can easily enter the bloodstream and metastasize. Clearly, angiogenesis is a process that contributes to the aggressiveness associated with cancer. The elucidation of mechanisms of action of pro-angiogenic agents continues to be an important step towards the design of anticancer drugs.

The currently utilized angiogenesis assays can be summarized into three major groups with subcategories, where the groups in vitro, ex vivo, and in vivo correspond to the type of experiment and the subcategory corresponds to the stage in the angiogenic process that the assay evaluates (Table 1). Each method included in the Table has specific advantages and disadvantages, for example, the in vitro cell counting technique is both time- and cost-efficient, yet fails to accurately reproduce the conditions that ECs experience in a living human. Thus, a combination of these assays is often necessary to acquire an ample amount of information regarding the entire process [8].

Lack of an ideal angiogenesis assay is inferred from the multiplicity and variety of mechanisms of the methods presented above because an ideal assay would render a majority of these assays obsolete. An ideal assay would perfectly mimic a functioning human vascular microenvironment, have a transparent exterior for easy visualization or imaging of vascularization, and be easily reproducible and relatively inexpensive. Also, assays should reflect the actions of vascular growth factors as well as the pro-angiogenic activities of small molecules such as thyroid hormone, bradykinin, and angiogentensin II [9].

## 2. In Vitro Assays

A large variety of in vitro assays can be used to investigate the mechanisms of angiogenesis in simulated environments. The most successful of these assays attempt to emulate the conditions found within the organism of interest, although perfect reproduction of the human microenvironment is impossible. Therefore, due to the ease of acquisition of these cells, many angiogenesis assays employ ECs such as bovine aortic endothelial cells (BAECs), human umbilical vein endothelial cells (HUVECs), or human dermal microvascular endothelial cells (HDMECs) [10,11]. However, ECs are not homogeneous, and small differences between the cellular anatomies could cause large differences in angiogenic activity. Similarly, ECs in vivo experience different physiological forces that cannot be mimicked in vitro. In other words, an experiment conducted in vitro comes with a trade-off: the result of an in vitro analysis may prove efficient and insightful, yet unavoidably inconclusive. Therefore, the assays described in this section ought to be implemented to obtain initial information about potential pro- or anti-angiogenic agents. Standardized EC preparations and standardized media are commercially available, e.g., from Lifeline Cell Technology (Frederick, MD, USA) or American Type Culture Collection (ATCC, Manassas, VA, USA), and these may be helpful in comparing assay results from several laboratories. Multiple passages of stored ECs should also be understood to be a possible variable in cell behaviors, including responses in assays to pharmacologic or biologic probes.

### 2.1. Proliferation

An initial consideration in the use of a proliferation assay is whether the biomolecule to be tested is pro-angiogenic or anti-angiogenic. If the molecule is projected to be pro-angiogenic, a low basal or control rate of vessel formation in the assay is useful in detecting and quantitating the effect on angiogenesis. If the molecule is anti-angiogenic, then a high basal rate of vessel formation in the assay affords ease of detection and statistical quantitation of the effect.

Proliferation assays often provide the quickest results that are easy to interpret, but they do not measure every vital step in the angiogenesis process. While ECs could be rapidly multiplying and generating new blood vessels, there could also be a deprivation of another growth factor that triggers them to migrate and/or form new vessels. Therefore, an in vitro EC proliferation assay should be used in tandem with another assay that evaluates the migratory or formative stages in the angiogenic process to obtain sufficient information.

#### 2.1.1. Cell Counting

The simplest technique used to measure the proliferation of ECs is a direct cell count. In this method, ECs are harvested and grown in culture before being labeled with an organic dye such as trypan blue and exposed to potential angiogenic agents [10]. Although this method approximates proliferation, it only requires a hemocytometer and a light microscope, which renders this method both economical and easy to reproduce for analyzing rates of angiogenesis. The accuracy of this approximation can be enhanced through cell counting devices such as electronic Coulter counters, although this analysis will require ECs to be treated with trypsin first [10]. A problematic aspect of this assay is that the cells in vitro will be in a proliferative state as opposed to the normal, predominantly quiescent state of ECs in a living human. However, the hemocytometer is time-consuming, more prone to sampling error, and can only measure the high density of cells; therefore, other fluorescence-based kits (e.g., CyQuant) and automated platforms such as plate cytometers can be used instead for analyzing cell proliferation [8]. Other methods have also been developed to circumvent the tediousness of counting cells, including colorimetric assays and DNA synthesis assays [10,11,12,13], described next.

#### 2.1.2. Colorimetric Assays

Colorimetric assays take advantage of the natural processes occurring during cellular division and expose ECs to biomolecules that can be altered during cellular metabolism. Because the biomolecule will be processed by ECs, a byproduct of that metabolism will be left behind, and the amount of that byproduct will be directly proportional to the population of ECs. Therefore, this byproduct can be collected to give information on how the cell population is changing over time. The most commonly used colorimetric assay utilizes the biomolecule 3-(4,5-dimethylthiazol-2-yl)-2,5-diphenyltetrazolium bromide, known as MTT, which is metabolized by mitochondria in living cells; this means that a larger cell population will reduce MTT faster [14]. MTT in particular is widely used in colorimetric assays because it is yellow before it is broken down into a purple formazan [12,13]. The formazan dye produced by the ECs is dissolved in a solvent system that is then tested for absorbance with a spectrophotometer [10]. Recently, MTT assay results showed that a potential hypoxia-inducible factor-1 (HIF-1) inhibitor that limits angiogenesis did not disrupt living ECs [12], while other MTT assay results elucidated the strong anti-angiogenic properties of certain thiabendazole derivatives on multiple ECs [13].

MTT assays make it facile to evaluate cell viability based on the absorbance of the resulting formazan solution. However, the disadvantage of this technique is that the metabolic processes of ECs can be affected by the same angiogenic agents that are being evaluated. In other words, it cannot be concluded with absolute certainty that a change in proliferation of the ECs occurred because of angiogenesis-specific effects of the drug or metabolic effects of the agents unrelated to blood vessel formation. Also, because this method depends on the metabolism of living cells, it gives no information on the cytotoxicity of the drug itself because there is essentially no way of measuring the extent of apoptosis due to exposure to a potentially toxic chemical as opposed to apoptosis due to metabolic processes. Therefore, it may be worthwhile to confirm the results of an MTT assay using another angiogenesis assay.

#### 2.1.3. DNA

Similar to the colorimetric assays, DNA synthesis assays also take advantage of the cellular activity of ECs to measure proliferation. For successful proliferation, the rapid mitotic division of ECs requires a replication of the DNA of the parent cells. During this synthesis of new DNA, either tritiated thymidine (^3^H) or bromodeoxyuridine (BrdU) can be introduced to the system as a way of measuring cellular reproduction [11]. The tritiated thymidine, which is radioactive, will be incorporated into the new DNA. Therefore, a measure of the radioactivity of the newly formed DNA acts as an indicator of how many new cells were produced [11]. BrdU works in a similar fashion because BrdU contests with thymidine for inclusion in the DNA sequence. The difference in this method is that BrdU is non-radioactive, which makes this method more environmentally friendly. However, the trade-off with this assay comes in the method of detecting BrdU because it requires immunohistochemical techniques, which can prove costly, and results can be difficult to interpret accurately [10].

### 2.2. Migration

After proliferation and the deterioration of the basement membrane, ECs migrate away from their origin. All angiogenic models involve the sprouting of ECs from a monolayer, and the movement of ECs through the basement membrane is often referred to as “sprouting” and occurs due to nearby chemical signals that either promote or prevent angiogenic activity. Thus, the assays described in this section focus specifically on elucidating the motion of ECs upon exposure to certain biomolecules. This step in the process of angiogenesis is arguably the most important in cancer research because cellular migration is essential for metastasizing and achieving malignancy for most tumors. The ideal migration-based assays will reveal pertinent information about cell motility such as gradient effects, concentration effects, directional effects, and effects on the rate of migration.

#### 2.2.1. Wound Healing

Wound healing is one of the best, yet simplest examples of angiogenesis in action. In this assay, ECs are grown in culture until they have formed a confluent monolayer. Then, a “wound” is inflicted on the monolayer with a pipet tip, cell scraper, or other hard object. In response to the damage, nearby ECs will begin to proliferate and migrate to heal the wound. However, proliferative effects must be eliminated to study the migration of these cells exclusively. Therefore, typically a specific biomolecule is introduced to prevent proliferation. Although this method is mostly qualitative, data such as how far the cells move, how fast the cells move, and the area traveled through migration can all be evaluated quantitatively. This assay is relatively simple compared to other migration-based assays, but achieving reproducibility proves difficult nonetheless. The issues with reproducibility arise from inconsistencies in confluency as well as data that can be inherently difficult to measure accurately [10,12].

#### 2.2.2. HDMEC Sprouting

The sprouting of ECs represents another significant step in the process of angiogenesis, and many assays have been developed to monitor this spread of ECs. In order to model accurately the sprouting of vascular networks that occurs in vivo, HDMECs are suspended in fibrin gels and exposed to angiogenic growth factors. For best results, HDMECs are often grown to confluence on microbeads and then subsequently suspended within the fibrin. One method that reported success used HDMECs grown on Cytodex-3 microbeads, which were then treated with human serum and placed in fibrin [15,16]. This procedure produces a system that can be evaluated for effects on angiogenesis within 48 hours and has previously been used to study the anti-angiogenic capabilities of tetraiodothyroacetic acid (tetrac). The sprouting that occurs in this type of assay is usually graded microscopically, sometimes limiting the utility of statistical analysis. However, new vessels should be visible enough for simple counting and length measurements, which can be translated into useful data. A disadvantage of this method is the less than ideal materials used to represent the extracellular matrix and the basement membrane [10].

#### 2.2.3. Matrix Degradation

Vessel sprouting, which is formation of new blood vessels from a pre-existing vascular network, occurs after ECs interrupt the integrity of the basement membrane. This vessel sprouting requires degradation of both the laminin-rich basement membrane and proteolysis of the collagen-rich extracellular matrix. Sprouting requires EC proteolysis in the extracellular matrix by releasing proteases such as matrix metalloproteinases (MMPs) and aminopeptidases [10]. Because MMPs have been shown to play a significant role in degrading the extracellular matrix, angiogenesis assays have been developed to test for their activity. One such assay that can be used to test MMP activity is called the zymogen assay. This assay allows MMPs and other proteases to deteriorate an artificial surrounding matrix in order to determine the speed and amount of proteases present. To accomplish this, lysates or supernatants from ECs are taken to gather proteases and then passed through the artificial matrix using electrophoresis. Artificial matrices can be composed of a number of materials including collagen. The up side of using this method is that it is inexpensive and yields basic information regarding the activity of MMPs. The down side is that this method takes time and is difficult to prepare for multiple tests [10].

The list of pro-angiogenic factors (cytokines that enhance EC basement membrane degradation, proliferation, motility, and differentiation), is extensive. Not surprisingly, several of the molecules implicated also have roles in promoting EC MMP production.

#### 2.2.4. Boyden Chamber

In 1962, Stephen Boyden attempted to study the chemotaxis—the process of directional cell motility due to a chemical gradient—of ECs through a membrane to thoroughly investigate the effects of potential angiogenic factors [17]. His original assay has been improved since then, but its variations are still commonly referred to as the modified Boyden chamber assay or the Trans well assay. In a sense, his original study adapted the way scientists used to investigate the diffusion of liquids through a membrane; one half of the chamber contains ECs, the other half contains a biomolecule being tested for angiogenic effects, and the central filter facilitates migration between the two sections. In an effort to model migration through the extracellular matrix, the porous filter is often coated with collagen, fibronectin, or some other matrix protein that ECs can bind to effectively, because cell movement needs a certain level of adhesion [10]. The ECs are then grown on the coating of the filter, and the growth factor under investigation is placed on the opposite side of the membrane. The filter should have pores with diameters 3–8 µm to allow for EC migration. After a short time (<24 h), the ECs will have migrated in response to the chemical stimuli, and the number of cells that passed through the membrane can be counted after being dyed. The speed of this assay is one of the advantages of this method, along with its sensitivity to changes in chemical concentration [18]. However, the two main disadvantages of this technique are that the filters coated with matrix proteins are often expensive relative to other angiogenesis assays and the assay system is difficult to maintain; the filter allows chemotactic agents to pass through the membrane so that the chemical gradients will eventually equilibrate throughout the chamber and cause random motion of cells (chemokinesis) rather than the desired chemotaxis [10,11,17,19].

#### 2.2.5. Phagokinetic Track

The migration of ECs can be quantified easily using a phagokinetic track assay. In this technique, ECs are plated on a substrate that has been covered with gold nanoparticles and a chemical stimulus will either promote or hinder motility. Movement of the ECs will force the colloidal gold to be moved aside or be absorbed by the cells due to the enhanced permeability and retention (EPR) effect attributed to gold nanoparticles. Because the gold nanoparticles will adjust as a result of EC motion, the empty space can be evaluated to obtain data regarding the direction and total movement of the cells. The disadvantage here is that a colloidal gold substrate is essentially a foreign construct that does not accurately reflect human physiology [11].

### 2.3. Tube Formation

Tube formation assays are commonly used for studying the process of angiogenesis. They examine the critical step of forming new capillaries that is so important to angiogenesis such that the entire process is ultimately dependent on the morphogenesis of ECs into new blood vessels. Therefore, tube formation assays are designed with the aim of identifying biomolecules that disrupt EC differentiation, and biomolecules that are successful often have the most potent anti-angiogenic properties.

#### 2.3.1. Matrigel

Tube formation, also known as tubulogenesis, connects existing vasculature to new blood vessels through the extracellular matrix. To model this process in vitro, ECs such as HUVECS or HDMECs are grown in culture and then placed on Matrigel^®^ to examine their angiogenic properties. Matrigel assays are typically two-dimensional imitations of the extracellular matrix found in living systems and are composed of extracellular matrix material and basement membrane proteins from Engelbreth–Holm–Swarm mice sarcoma [20]. The Matrigel acts as a substrate for ECs, which can then form tubules due to the presence of cytokines and other growth factors [11]. Tube formation can be observed with imaging techniques and may take as little as a day to provide reliable data. However, it has been shown that fibroblasts and other non-ECs can produce vessels/networks on Matrigel assays. Similarly, ECs can produce “bridges” to other cell types instead of forming lumens that control blood vessel formation during development and postnatal life [21]. Therefore, reliable data can only be obtained if samples of ECs plated on Matrigel are entirely void of fibroblasts and/or cancerous cells. In this method, verification that lumens are present within the capillary-like structures may also be necessary.

While Matrigel assays are typically two-dimensional, they can be constructed to mimic angiogenesis in three dimensions as well. Fibrin clots, or even layers of collagen, can also be used for a more representative, three-dimensional analysis [22]. To accomplish this, ECs are grown nearly to the point of confluence and stuck between layers of extracellular matrix components to form a substrate in more than two directions. Tubules generally form horizontally between the layers, but vertical penetration of the ECs through the layers is possible depending on the presence of growth factors [23]. The main advantages of this technique are the accurate representation of a three-dimensional substrate and the ability to evaluate potential pro-angiogenic factors. However, a disadvantage is that an analysis on tube formation in three dimensions can be arduous and time-consuming.

#### 2.3.2. Co-Culture

Co-culture assays provide a two-fold solution to evaluating angiogenesis. They are used to investigate specific cell lines as well as specific biomolecules like VEGF for their effects on ECs’ behavior. For example, ECs can be grown and then exposed to fibroblasts, cancer cells, muscle cells, or explants composed of many types of cells to see what kind of effects are elicited. At the same time, growth factors can be introduced or eliminated to see additional effects. One simple co-culture model involves a two-chambered well containing ECs (often HDMECs) and tumor cells, separated by a porous polycarbonate membrane. The HDMECs are often grown on Matrigel with reduced growth factor expression, while the cells under investigation are plated on type I collagen. The contents of both chambers can be manipulated up until the point of co-culture to test for other effects. Upon co-culture, ECs will exhibit sprouting and capillary formation towards a stimulant or reduced activity in the presence of an inhibitor. To evaluate the tubulogenesis of this assay, HDMECs are stained with a CD31/PECAM antibody specific for the binding of ECs, which are then imaged microscopically. The main advantage of this method, like the Matrigel assay, comes from a more reliable simulation of in vivo conditions. However, this method is time-consuming, and could take up to two weeks to set up the apparatus and recover data.

## 3. Ex Vivo Assays

### 3.1. Thoracic Aorta Ring (Mouse and Rat)

The three-dimensional ex vivo thoracic aorta ring (TAR) model combines the advantages of in vitro and in vivo models and is one of the most popular in vitro methods for studying angiogenesis. As the name suggests, this assay requires thoracic aorta ring from mouse or rat, excised and cut into multiple sections (~1 mm) and immersed in gels comprised of collagen or fibrin [10,11]. The rings are then cultured in serum-free media, permitting vessel sprouting [24]. After a few days, the outgrowth can be measured using staining and microscopy, then compared to samples that have been exposed to potential angiogenic agents. The advantages of the TAR assay are numerous. First and foremost, the TAR assay was developed to investigate the effects of angiogenic agents on transgenic animals. For example, the outgrowth imaging for a mouse whose ECs express green fluorescent protein is much simpler, and it allows one to circumvent immunological staining [10]. The TAR assay is also relatively easy to reproduce and provides a realistic simulation of the conditions in an intact animal. However, imprecise cuts of the adventitia and varying aorta sizes in mice may lead to difficulties in using this assay.

Recently, the rat TAR assay was used to show the anti-angiogenic properties of two thiabendazole derivatives [13]. Thiabendazole is an FDA-approved, orally administered drug that treats both fungal and parasitic infections. An altered form of thiabendazole named TBZ-19 was shown to be an effective inhibitor of cell proliferation and vascularization through visualization of rat thoracic aorta rings, which displayed substantial outgrowth in untreated rings but limited outgrowth in rings treated with TBZ-19 [13].

### 3.2. Ex Vivo Retina Angiogenesis

The ex vivo retina model of neovascularization will decrease the number of animal experiments needed by obtaining several retinal fragments from the eye of one animal. In this assay, isolated retina fragments from mice are embedded in a three-dimensional fibrin gel containing VEGF, which plays a critical role in retinal neovascularization, and then the fragments are incubated for 3–4 days. The EC sprouts will invade into the fibrin gel and can easily be observed with upright fluorescence microscopy. VEGF’s effect is dose-dependent and maximal stimulation can be observed between 6–14 days [25]. The main advantages of this technique are the precise evaluation of vascular sprouting from mature vessels in the adult retina and the ability to evaluate potential pro-angiogenic factors. A disadvantage of this model is the lack of blood flow, circulating endothelial progenitors, and hormonal factors that a have key role in angiogenesis [26].

## 4. In Vivo Assays

Assessment of angiogenesis in vitro often provides efficient results, but these results will not necessarily translate to intact biological systems. ECs are heterogeneous, and cell media conditions between studies are rarely identical, which leads to more discrepancies.

In vivo assays provide the most reliable information on how a biomolecule may affect the process of angiogenesis in an intact organism and are essential for the development of anti- or pro-angiogenic agents. The most commonly used in vivo assays for studying angiogenesis are described next and should be used as final confirmation of the results of in vitro investigations.

### 4.1. Chick Chorioallantoic Membrane

The chick chorioallantoic membrane (CAM) assay is one of the oldest and most widely used assays for studying angiogenesis in vivo. It was developed by Folkman et al. and exploits the fact that chorioallantoic membranes are present in the fertilized eggs of all avian species and contain a copious number of blood vessels [10,11]. These characteristics are ideal for in vivo assays because chicken eggs are inexpensive, the method is easy to reproduce technically and suits large-scale screening, and visualization of new vascularization is simple under a microscope. Also, in terms of ethics, this assay is one of the more acceptable methods of investigating angiogenesis in vivo. However, the CAM assay has one main disadvantage, namely, its dense web of blood vessels makes it difficult to use for evaluating weakly pro-angiogenic agents.

The first step in this technique is to locate an avascular area in the membrane, which is accomplished by shining light through the egg shell. To access the membrane, a small “window” is cut into the shell, and the cut-out piece of shell can be replaced because the membrane is highly sensitive to oxygen exposure. However, initial removal of this section of egg shell could stimulate an inflammatory response and make visualization difficult. For this reason, it is often necessary to wait a few days before adding the potential angiogenic agent to the CAM. After that preparatory period, the test agent is introduced to the CAM using slow-release pellets, filter discs, or xenografts. After a few more days, the region of the membrane under investigation is excised from the egg and imaged for new vascularization. An alternative CAM method relocates the entire embryo from the shell to a petri dish in culture before the angiogenic agent is introduced. This method often produces better data because vascularization occurs in a larger, more readily accessible area.

The CAM assay has been used extensively to define the pro-angiogenic effects of VEGF and anti-angiogenic actions of the small molecule tetrac, a thyroid hormone derivative. The VEGF protein and tetrac interact with discrete sites on the extracellular domain of EC integrin αvβ3. Tumor xenografts in the CAM also permit study of tumor-relevant ECs. The CAM assay has generated information about the inhibitory actions of tetrac on angiogenesis regulated by VEGF and a panel of other vascular growth factor proteins and small molecule angiogenesis regulators.

### 4.2. Zebrafish

The zebrafish is optimal for assessing angiogenesis in vivo for a variety of reasons. Most importantly, the zebrafish can produce hundreds of embryos per week through mating. The large number of embryos produced each week are yolk sac-dependent, making this type of assay perfect for large-scale screening. Because the embryos are transparent, vascularization in the yolk-sac is easily viewed through a microscope. Recently, zebrafish embryos were genetically engineered to enable blood vessels to exhibit green fluorescent protein; blood cells exhibit a different fluorescent color, making visualization of new blood vessel formation and blood flow even easier [27]. The zebrafish assay model can also be useful to identify genetic factors involved in angiogenesis. For example, gene knockdowns can be performed with siRNAs to determine which genes play a predominant role in the angiogenic process. The extent of angiogenesis can then be assessed with angiography or confocal microscopy. The main concern with this assay is the type of vascularization that occurs. Because embryos are employed in this model, there must be a well-defined distinction between angiogenesis and vasculogenesis [28].

### 4.3. Corneal Angiogenesis

The transparent front circular structure of the eye, the cornea, presents a nearly ideal region for evaluating angiogenesis in vivo. Notably, the cornea does not contain any pre-existing vasculature, so any new blood vessels that arise in this region must be a result of angiogenesis [29]. Also, the cornea is transparent, so this vascularization is easily visualized. This assay manipulates these properties of the cornea by creating what is termed a stromal “pocket” immediately behind the cornea and subsequently filling that pocket with the angiogenic agents under investigation (cancer cells, VEGF, thyroid hormones, etc.). These agents are typically delivered via slow-release pellets or sponges to monitor the progression of angiogenesis daily [11]. To quantify the data, new vasculature can be stained and images of the outgrowth can be analyzed with computer programs. Corneal angiogenesis can also be done using the alkali-burn injury corneal neovascularization model in the mouse, which can be instrumental to study the mechanisms for intervention in neovascular disorders and to evaluate therapeutic effects of the histone deacetylase (HDAC) inhibitors in the pathogenesis of corneal neovascularization [30].

The corneal angiogenesis assay does not come without drawbacks, however. It requires the manipulation of an important organ of a living animal, and thus, the major issue with this assay will be an ethical one. Secondly, although evaluation of the cornea proves reliable, the cost and difficulty of this assay is much greater compared to the CAM and zebrafish assays. For this assay to be ideal, the cornea must be generated from an external source (or cause no pain to the animal), and the methods used to create stromal pockets must be improved.

### 4.4. Matrigel Plug

The Matrigel plug assay integrates the previously described in vitro EC substrate Matrigel as an in vivo tool for evaluating angiogenesis. Matrigel is chemically unusual in that it exists as a liquid at cold temperatures (4 °C) and undergoes a phase change to solid upon reaching 37 °C. In this assay, cold Matrigel is injected subcutaneously, usually into a rat or mouse. The Matrigel then begins to form a solid plug after just half an hour, and angiogenic agents are generally inserted into the Matrigel matrix by means of slow-release pellets or sponges. After an incubation period that typically lasts a few days, the Matrigel plug is excised and immunohistologically stained for imaging. The vascular growth that extends into the plug can then be measured by quantifying the CD31-positive vessels or amount of hemoglobin present [11,31,32]. This assay represents one of the most realistic in vivo methods because the Matrigel closely imitates the extracellular matrix of living systems. However, as previously stated, Matrigel is fairly expensive, and the use of non-identical sponges containing slow-release pellets could lead to systemic error in the data. Regardless, Matrigel plugs have become one of the most common methods of investigating the extent of angiogenesis in living systems.

### 4.5. Xenograft

Xenograft assays provide another viable option for evaluating angiogenesis in vivo. In this method, a foreign cell line is injected into an immunodeficient host organism to form a pathologic entity such as a cancer. In most cases, the role of the host organism belongs to mice and rats while the foreign cell lines are often derived from human cells afflicted with a biological disorder under investigation. After the cells are injected into the host and display the desired characteristics, drugs are administered over time to determine the effects on the pathologic entity in vivo. The xenografted tumor can then be removed and analyzed with single photon emission computed tomography (SPECT) [33], confocal fluorescence microscopy [34], or immunohistochemical staining [35]. Xenograft assays provide a method of evaluating the effect of a drug on the angiogenic process and the pharmacokinetics of the drug itself, making this assay suitable for most long-term drug studies.

Recently, the xenograft model was used to study the effects of melatonin on a transplanted human breast adenocarcinoma cell line in mice [33]. MDA-MB-231 cells were injected subcutaneously into the hind flank of immunodeficient mice to generate a tumor and, consequently, an angiogenic response. Melatonin was then delivered to the mice intraperitoneally and the size of the tumors was monitored weekly using a digital calipers. Although the xenograft technique requires expertise, it produces angiogenic and pharmacokinetic data that can be evaluated over an extended time period.

## 5. Conclusions

Research on angiogenesis has tremendous implications for the field of cancer therapy and the treatment of ischemic diseases. The discovery and identification of agents that selectively halt the process of angiogenesis could eliminate the risk of metastatic tumors dramatically. Conversely, the elucidation of pro-angiogenic factors could alleviate some of the dangers associated with ischemia (restriction in blood supply to tissues) and play a central role in novel therapies that promote blood flow through vascularization. Clearly, angiogenesis assays are the toolkit for understanding this complex process of new blood vessel formation, and the development of enhanced evaluation methods is crucial in order to eliminate errors due to non-ideal microenvironments and other external variables.

## Figures and Tables

**Table 1:** Angiogenesis assays: the most commonly used methods to evaluate angiogenesis modulators.

**In Vitro Assay**	**Technique**	**Advantages**	**Disadvantages**
**Proliferation**	Cell counting	→ Low cost	→ High human error → Requires high number of cells and multiple counts to achieve accuracy
	Colorimetric	→ Easy to use, low cost, safe, high reproducibility → Used to determine both cell viability and cytotoxicity → Potential for automation	→ Toxic side effects of some dyes on mammalian cells → Time consuming → Contamination of reusable cell counting chambers
	DNA synthesis	→ Potential to measure accurately toxicity of the biomolecule by evaluating extent of apoptosis	→ Relatively high cost of immunohistochemical techniques → Difficult to interpret results accurately
**Migration**	Wound healing	→ Simple and qualitative compared to other migration-based assays	→ Difficult to achieve reproducibility → Inconsistencies in confluency and data → Difficult to interpret results accurately
	Human dermal microvascular endothelial cell (HDMEC) sprouting	→ Can evaluate effects on angiogenesis within 48 h → Robust, reproducible, and representative model of microvascular angiogenesis → Semi-automated software for quantification of sprouting area is available	→ Less than ideal materials used to represent the extracellular matrix and the basement membrane
	Matrix degradation	→ Inexpensive → Easy to get basic information	→ Time consuming → Difficult to prepare for multiple tests
	Boyden chamber	→ Fast → Sensitive to changes in chemical concentration	→ Expensive → Difficult to maintain
	Phagokinetic track	→ Quick, quantitative, easy measure of cellular motility → Simple high-throughput assay, for use with cell types that are not amenable to time-lapse imaging	→ The colloidal gold substrate used is essentially a foreign construct that does not accurately reflect human physiology
**Tube Formation**	Matrigel	→ Accurate representation of a three-dimensional substrate → Can evaluate potential pro-angiogenic factors	→ Time-consuming → Technically difficult
	Co-culture	→ Tubules form lumen → Reliable	→ Time-consuming (up to two weeks to set up apparatus and recover data)
**Ex Vivo Assay**	**Technique**	**Advantages**	**Disadvantages**
**Thoracic Aorta Ring**		→ Easy to reproduce → Realistic simulation of conditions in intact animals	→ Technically difficult because of imprecise cuts of the adventitia and varying aorta sizes in mice
**Retina model**		→ Precise evaluation of vascular sprouting from mature vessels in the adult → Can maintain retinal vessel architecture and assess contribution of other cell types to the growth of new vessels	→ Isolation of the retina is a critical step, requiring careful handling of the specimen → Age of mice or other animal sources may be a critical factor → Lack of blood flow, circulating endothelial progenitors, and hormonal factors that have key role in angiogenesis
**In Vivo Assay**	**Technique**	**Advantages**	**Disadvantages**
**Chick Chorioallantoic Membrane**		→ Low cost of chicken eggs → Easy to reproduce technically and suits large-scale screening → Easily visualized under a microscope	→ Difficult to distinguish normal angiogenesis from induced
**Zebrafish**		→ Potential to produce large number of yolk sac-dependent embryos for large-scale screening → Relatively quick assay (6–12 h) and easily visualized under a microscope → Disruption to vasculature does not damage the embryo	→ Does not distinguish the exact point in angiogenic cascade specifically disrupted → Cannot distinguish between angiogenesis and vasculogenesis
**Corneal Angiogenesis**		→ Repeatable method that can be applied in different animal models including rabbit, mouse, rat, guinea pig → Easy identification of new blood vessels → Reliable results because the cornea does not contain any pre-existing vasculature, so any new blood must be from angiogenesis	→ Expensive → Not suitable for large-scale screening → Occurs in a non-vascular environment
**Xenograft**		→ Suitable for most long-term drug studies	→ Requires expertise and cost to evaluate the data over extended time periods
**Matrigel Plug**		→ Most realistic for in vivo angiogenesis → Technically not difficult → Quantification can be done by measuring the amount of hemoglobin in the plug	→ Fairly expensive → Possible to have some systemic error in the data because of non-identical sponges containing slow-release pellets

HDMEC, human dermal microvascular endothelial cells.

## References

[1] Srinivasan M., Rajabi M., Mousa S.A., Grumezescus A.M. (2016). Nanobiomaterials in cancer therapy. Applications of Nanobiomaterials.

[2] Rajabi M., Mousa S.A. (2017). The role of angiogenesis in cancer treatment. Biomedicines.

[3] Cook K.M., Figg W.D. (2010). Angiogenesis inhibitors: Current strategies and future prospects. CA Cancer J. Clin..

[4] Vasudev N.S., Reynolds A.R. (2014). Anti-angiogenic therapy for cancer: Current progress, unresolved questions and future directions. Angiogenesis.

[5] Welti J., Loges S., Dimmeler S., Carmeliet P. (2013). Recent molecular discoveries in angiogenesis and antiangiogenic therapies in cancer. J. Clin. Investig..

[6] Dvorak H.F., Brown L.F., Detmar M., Dvorak A.M. (1995). Vascular permeability factor/vascular endothelial growth factor, microvascular hyperpermeability, and angiogenesis. Am. J. Pathol..

[7] Hoeben A., Landuyt B., Highley M.S., Wildiers H., Van Oosterom A.T., De Bruijn E.A. (2004). Vascular endothelial growth factor and angiogenesis. Pharmacol. Rev..

[8] Nowak-Sliwinska P., Alitalo K., Allen E., Anisimov A., Aplin A.C., Auerbach R., Augustin H.G., Bates D.O., van Beijnum J.R., Bender R.H.F. (2018). Consensus guidelines for the use and interpretation of angiogenesis assays. Angiogenesis.

[9] Davis P.J., Sudha T., Lin H.Y., Mousa S.A. (2015). Thyroid hormone, hormone analogs, and angiogenesis. Compr. Physiol..

[10] Goodwin A.M. (2007). In vitro assays of angiogenesis for assessment of angiogenic and anti-angiogenic agents. Microvasc. Res..

[11] Staton C.A., Stribbling S.M., Tazzyman S., Hughes R., Brown N.J., Lewis C.E. (2004). Current methods for assaying angiogenesis in vitro and in vivo. Int. J. Exp. Pathol..

[12] Wei J., Yang Y., Li Y., Mo X., Guo X., Zhang X., Xu X., Jiang Z., You Q. (2017). Synthesis and evaluation of N-(benzofuran-5-yl)aromaticsulfonamide derivatives as novel HIF-1 inhibitors that possess anti-angiogenic potential. Bioorg. Med. Chem..

[13] Zhang C., Zhong B., Yang S., Pan L., Yu S., Li Z., Li S., Su B., Meng X. (2015). Synthesis and biological evaluation of thiabendazole derivatives as anti-angiogenesis and vascular disrupting agents. Bioorg. Med. Chem..

[14] Rajabi M., Hossaini Z., Khalilzadeh M.A., Datta S., Halder M., Mousa S.A. (2015). Synthesis of a new class of furo[3,2-c]coumarins and its anticancer activity. J. Photochem. Photobiol. B.

[15] Feng X., Tonnesen M.G., Mousa S.A., Clark R.A. (2013). Fibrin and collagen differentially but synergistically regulate sprout angiogenesis of human dermal microvascular endothelial cells in 3-dimensional matrix. Int. J. Cell Biol..

[16] Davis P.J., Davis F.B., Mousa S.A. (2009). Thyroid hormone-induced angiogenesis. Curr. Cardiol. Rev..

[17] Boyden S. (1962). The chemotactic effect of mixtures of antibody and antigen on polymorphonuclear leucocytes. J. Exp. Med..

[18] Falk W., Goodwin R.H., Leonard E.J. (1980). A 48-well micro chemotaxis assembly for rapid and accurate measurement of leukocyte migration. J. Immunol. Methods.

[19] Zigmond S.H., Hirsch J.G. (1973). Leukocyte locomotion and chemotaxis. New methods for evaluation, and demonstration of a cell-derived chemotactic factor. J. Exp. Med..

[20] Lawley T.J., Kubota Y. (1989). Induction of morphologic differentiation of endothelial cells in culture. J. Investig. Dermatol..

[21] Sacharidou A., Stratman A.N., Davis G.E. (2012). Molecular mechanisms controlling vascular lumen formation in three-dimensional extracellular matrices. Cells Tissues Organs.

[22] Montesano R., Pepper M.S., Orci L. (1993). Paracrine induction of angiogenesis in vitro by Swiss 3T3 fibroblasts. J. Cell Sci..

[23] Gagnon E., Cattaruzzi P., Griffith M., Muzakare L., LeFlao K., Faure R., Beliveau R., Hussain S.N., Koutsilieris M., Doillon C.J. (2002). Human vascular endothelial cells with extended life spans: In vitro cell response, protein expression, and angiogenesis. Angiogenesis.

[24] Nicosia R.F., Ottinetti A. (1990). Growth of microvessels in serum-free matrix culture of rat aorta. A quantitative assay of angiogenesis in vitro. Lab. Investig..

[25] Rezzola S., Belleri M., Ribatti D., Costagliola C., Presta M., Semeraro F. (2013). A novel ex vivo murine retina angiogenesis (EMRA) assay. Exp. Eye Res..

[26] Moleiro A.F., Conceicao G., Leite-Moreira A.F., Rocha-Sousa A. (2017). A critical analysis of the available in vitro and ex vivo methods to study retinal angiogenesis. J. Ophthalmol..

[27] Udvadia A.J., Linney E. (2003). Windows into development: Historic, current, and future perspectives on transgenic zebrafish. Dev. Biol..

[28] Gore A.V., Monzo K., Cha Y.R., Pan W., Weinstein B.M. (2012). Vascular development in the zebrafish. Cold Spring Harb. Perspect. Med..

[29] Gimbrone M.A., Cotran R.S., Leapman S.B., Folkman J. (1974). Tumor growth and neovascularization: An experimental model using the rabbit cornea. J. Natl. Cancer Inst..

[30] Anderson C., Zhou Q., Wang S. (2014). An alkali-burn injury model of corneal neovascularization in the mouse. J. Vis. Exp..

[31] Rajabi M., Sudha T., Darwish N.H., Davis P.J., Mousa S.A. (2016). Synthesis of MR-49, a deiodinated analog of tetraiodothyroacetic acid (tetrac), as a novel pro-angiogenesis modulator. Bioorg. Med. Chem. Lett..

[32] Rajabi M., Yalcin M., Mousa S.A. (2018). Synthesis of new analogs of tetraiodothyroacetic acid (tetrac) as novel angiogenesis inhibitors for treatment of cancer. Bioorg. Med. Chem. Lett..

[33] Jardim-Perassi B.V., Arbab A.S., Ferreira L.C., Borin T.F., Varma N.R., Iskander A.S., Shankar A., Ali M.M., de Campos Zuccari D.A. (2014). Effect of melatonin on tumor growth and angiogenesis in xenograft model of breast cancer. PLoS ONE.

[34] Chiavacci E., Rizzo M., Pitto L., Patella F., Evangelista M., Mariani L., Rainaldi G. (2015). The zebrafish/tumor xenograft angiogenesis assay as a tool for screening anti-angiogenic miRNAs. Cytotechnology.

[35] Kodama A., Sakai H., Matsuura S., Murakami M., Murai A., Mori T., Maruo K., Kimura T., Masegi T., Yanai T. (2009). Establishment of canine hemangiosarcoma xenograft models expressing endothelial growth factors, their receptors, and angiogenesis-associated homeobox genes. BMC Cancer.

